# Large abdominal aortic aneurysm presented with concomitant acute lumbar disc herniation – a case report

**DOI:** 10.25122/jml-2021-0419

**Published:** 2022-06

**Authors:** Eric Chun-Pu Chu

**Affiliations:** 1 New York Chiropractic and Physiotherapy Centre, Hong Kong SAR, China

**Keywords:** AAA, chiropractic therapy, abdominal aortic aneurysms, patient-centric, AAA – Abdominal aortic aneurysm, MRI – Magnetic resonance image, NSAIDs – Nonsteroidal anti-inflammatory drugs, CT – Computed tomography

## Abstract

The purpose of this case report was to describe chiropractic management of acute lumbar disc herniation in a patient with a large abdominal aortic aneurysm. A 72-year-old male patient presented with low back pain and right lower leg numbness for 12 months. A review of full-spine X-ray and lumbar MRI revealed moderate spondylosis at L2-5, moderate lumbar scoliosis, and a 7.15 cm abdominal aortic aneurysm (AAA). Given the minimum 2-weeks of referral waiting time to receive treatment for AAA, the patient received chiropractic treatment with a hybrid rehabilitation to address the disc herniation causing severe physical disability. Through the treatments, the patient's pain was significantly alleviated with careful consideration of potential risk factors associated with AAA. In addition, the acute disc herniation was successfully managed by a series of chiropractic treatments before and after the operation for AAA. This case supports that low back pain in patients with AAA can be managed by manual therapy, in contrast to a widespread belief that manual therapy is contraindicated in AAA. More case reports of AAA patients with low back pain are warranted to assess the effectiveness and safety of manual therapy along with surgical treatment for AAA.

## INTRODUCTION

Abdominal aortic aneurysms (AAA) affect over 200,000 people annually only in the United States. Ultrasound screening studies revealed that 4–8% of the male geriatric population have an occult abdominal aortic aneurysm [1]. Frequently, it is identified coincidentally with other diagnostic techniques [2]. Ruptured aneurysms have a fatality rate of 50% to 95%, accounting for the 10^th^ leading cause of death in men older than 55 [3]. AAAs can be detected during a routine abdominal physical examination in which the physician palpates the abdomen for an enlarged pulsating mass more than 3 cm in diameter [2, 4]. However, the palpation examination suffers from a low sensitivity of less than 65% [3], and approximately 30% of ruptured AAAs are misdiagnosed [3]. The geriatric population in the United States was about 16.9%, and 5% of them see chiropractors annually [5]. Thus, it is estimated that a substantial portion of geriatric patients who presented to chiropractors may also have AAA.

Spinal manipulation performed by chiropractic professionals has traditionally been regarded as a red flag in patients with AAA despite the absence of published reports showing its correlation with aortic dissection [6–8]. It has been considered that increased intraabdominal or intrathoracic pressure during spinal manipulation may increase the likelihood of dissection or rupture of the aorta. This view is challenged by the paucity of chiropractic reports on patients with AAA since the last case in 2010 [9]. As such, this report aims to detail the chiropractic care provided to a patient with large AAA and acute lumbar disc herniation without a notable secondary complication.

## CASE REPORT

A 72-year-old Asian male experienced mild right wrist pain, moderate low back pain, and right lower leg numbness for 12 months. The symptoms first started as ache and constant low back pain for 2 years that slowly radiated as episodic right leg numbness for 12 months. As the symptoms had suddenly worsened over the past 2 months, the patient could walk only with a cane. He had difficulties walking for over 15 minutes and more frequent awakening at night due to back pain. The patient had a past medical history of angioplasty and gout 10 years ago, with no known trauma history. The severity of back pain was rated 9/10, which negatively impacted his quality of life and mental health. He was initially diagnosed by the family physician with sciatic pain and received treatment including NSAIDs and celecoxib (Celebrex^®^), which provided only temporary relief. The patient then sought chiropractic care for conservative management.

*Investigation*: Tophus formations were identified in bilateral hands, and lumbar scoliosis was observed with paraspinal muscle spasm. His pain was provoked by trunk extension, right bending, and right ankle inversion and eversion. The lower quarter neurological screening revealed a diminished light touch of the right ankle and feet. The spinal intervertebral restriction was identified at L4/5 and the right sacroiliac joint. His range of motion in the lumbar region was restricted by muscle spasms. The straight leg test was positive for disc herniation at 30°. He was sent to radiography in a weight-bearing position to evaluate his orthopedic conditions. To alleviate his symptoms, spinal manipulation with NYMG Scoliosis^®^ protocol was applied to his lumbar spine, and acupuncture was administered to the paraspinal muscles. The patient had immediate relief from his symptoms.

**Figure 1:**
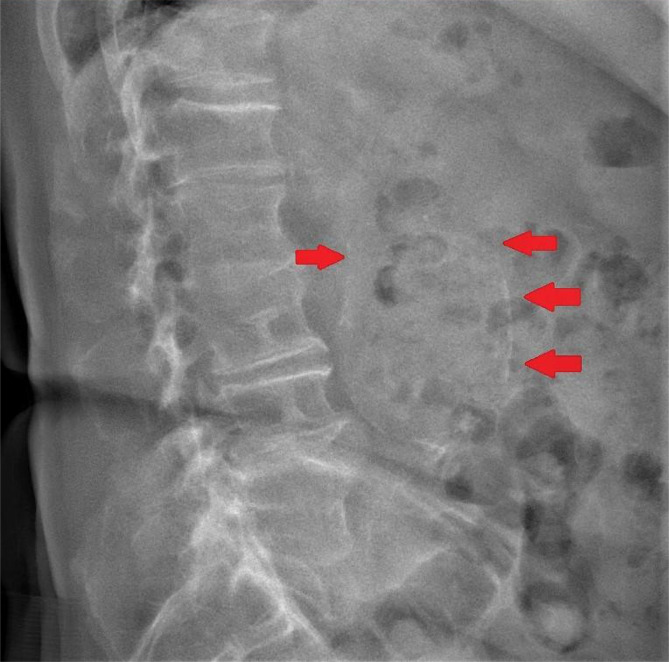
Lateral view identified the presence of a significant calcified aorta from the L2-L5 region.

The patient described the symptoms as reduced by 20% on the 2^nd^ visit, and his sleeping quality improved. The radiograph showed moderate spondylosis at L2-5, moderate lumbar scoliosis, and a presence of a significant calcified aorta from the L2-L5 region (Figure 1). An apparent increase in soft tissue opacity was observed over the volar aspect of the right wrist, consistently with gouty tophi deposition. With the differential diagnosis of gout, AAA, and lumbar disc herniation, the patient was arranged for a lumbar MRI and abdominal CT aortogram with the radiology department. Careful pretreatment planning was organized with the rehabilitation, radiology, and orthopedic departments. Hybrid conservative techniques, including acupuncture, chiropractic instrument adjustment technique, gua sha soft tissue treatment, joint mobilization, and preoperative physical therapy, were considered reasonable strategies for disc herniation. Any types of high-velocity spinal manipulation techniques were avoided.

**Figure 2:**
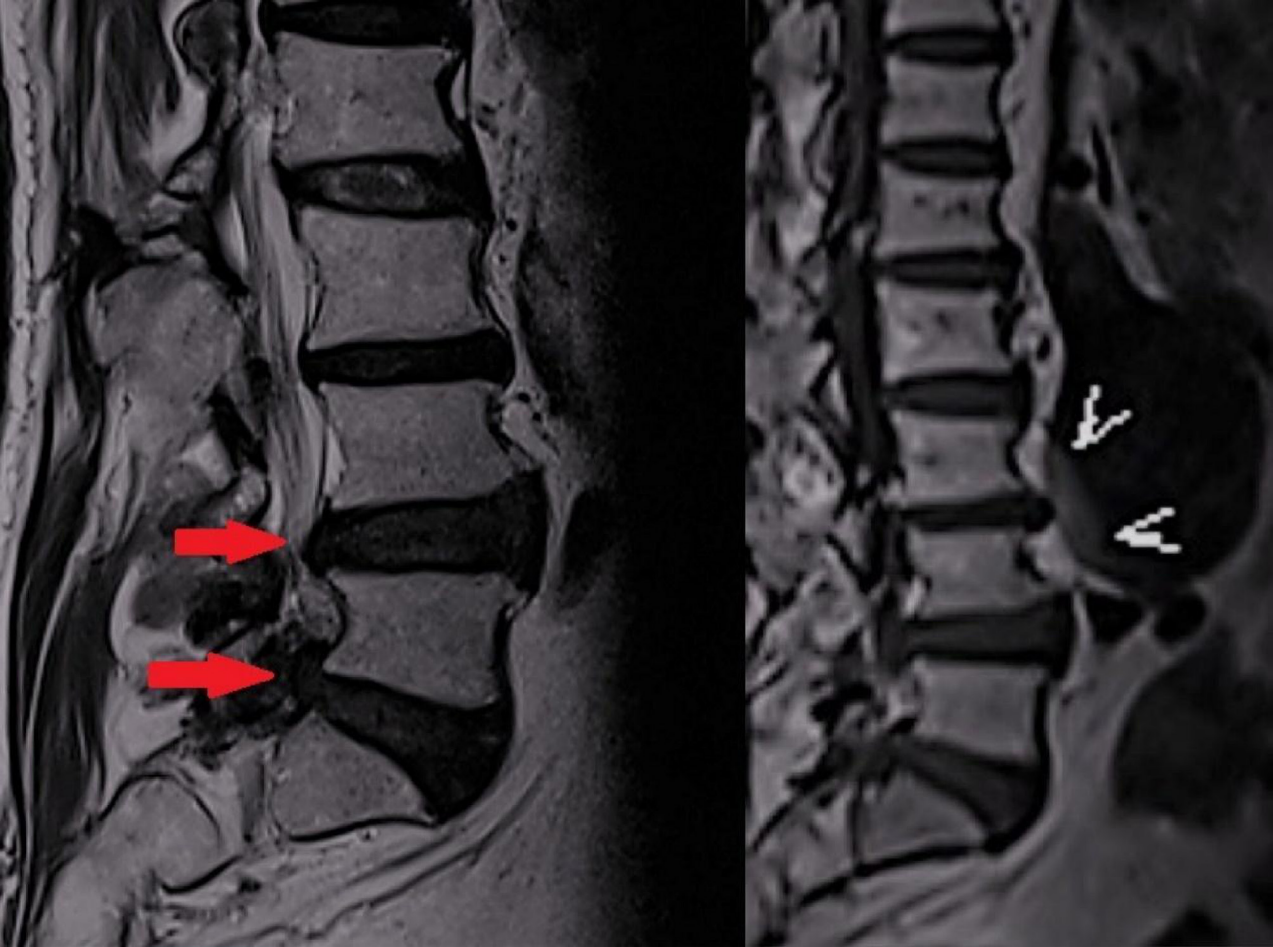
Magnetic resonance (MR) image revealed dilatation of abdominal aorta (red arrows) and marginal osteophyte formation of the lumbar vertebrae and posterior protrusion at the L3/L4 and L4/L5 levels pinching the spinal nerve extending from the spinal cord and cauda equina (red arrow).

**Figure 3:**
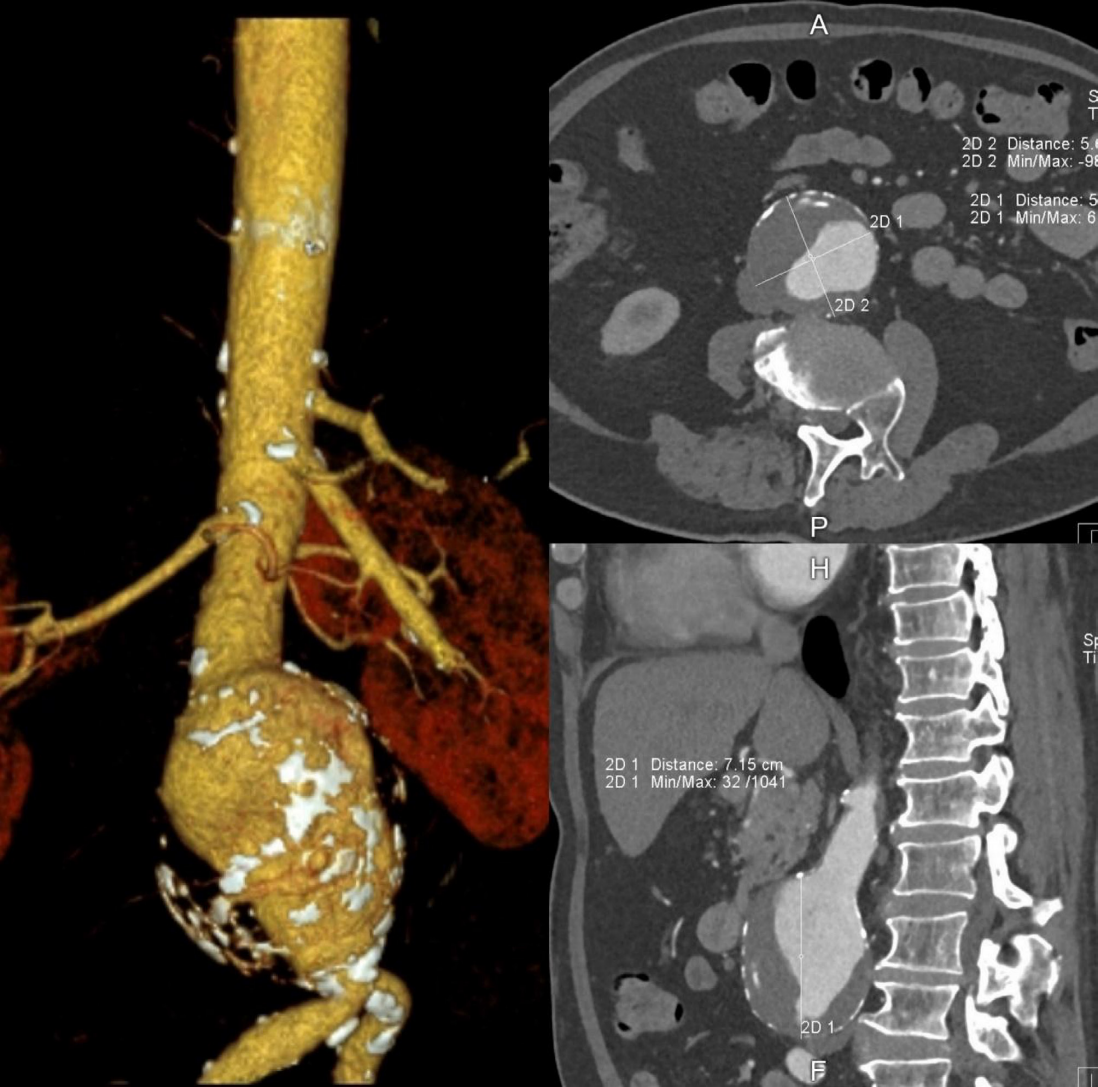
Computed tomography abdominal aortogram measured a 7.15 cm × 5.67 cm × 5.46 cm diameter abdominal aortic aneurysm.

The patient received conservative intervention daily for 5 consecutive days for the management of lumbar disc herniation. On the 6^th^ day of the clinic visit, his lumbar MRI identified disc bulging compressing on the spinal cord and cauda equina at L3/4 and L4/5 levels and impingement of right nerve roots. In addition, a 7 cm AAA was identified at L2-4 (Figure 2). The CT abdominal aortogram (Figure 3) also confirmed the measurement of 7.15 cm × 5.67 cm × 5.46 cm diameter in the diagnosis of AAA. He was immediately referred to the cardiologist.

*Diagnosis:* On the 7^th^ day, he was reviewed by a cardiologist with the confirmation of lumbar disc herniation with an abdominal aortic aneurysm.

*Treatment:* He transferred to a vascular-surgical consultation on the 9^th^ day and received endovascular repair at the AAA and angioplasty of two of the coronary arteries on the 14^th^ day. At one week post-surgery, the patient started a post-operative physical therapy program with a cardiovascular fitness exercise prescription for 45 minutes at a target intensity of 60% heart rate reserve. Treatment for disc herniation and lumbar scoliosis was also administered three weeks after the surgery. The first phase of treatment aimed at regaining muscle strength and reducing back pain conservatively. Laser acupuncture and diversified chiropractic adjustment were applied 5 times a week for 2 weeks. A spinal decompression treatment by intermittent robotic traction (MID Series, WIZ Medical, Korea) was further added to the subsequent treatments to restore nerve dysfunction. The frequency of treatments was reduced to 3 times a week for 3 months.

*Outcome:* Under conservative management, the patient reported 50% relief of musculoskeletal symptoms before the operation. After the operation, the back pain and motor strength started to recover following the second week of post-surgical rehabilitation. The patient continued his treatment once a week, which focused on restoring lumbar disc herniation for an additional 3 months. His sensorimotor dysfunction was mostly resolved after treatment. He was symptom-free and was able to return to normal daily activity. The Oswestry Disability Index was reduced from 68% to 2%. At 12^th^ month follow-up, the patient remained healthy and returned to his weekly hiking hobby. No adverse event was reported.

## DISCUSSION

According to the literature study on PubMed, the last chiropractic case report of patients with AAAs was dated 11 years ago, and the published reports are mostly focused on the physical examinations without manual therapy when patients need surgical treatment for AAA [6, 8, 10–12]. As AAA is associated with 91% of low back pain [12], it is important to understand the procedures of co-management of back pain and AAA through multidisciplinary approaches. This is the first case report describing the clinical management of low back pain with co-management procedures for AAA from a patient-centric perspective. Most clinicians ignored the patient's original chief complaints when the AAA was identified. Most reports failed to describe the differentiated diagnosis with advanced imaging systems, collaborating treatment options with the surgical team, and following up on postoperative rehabilitation as the primary care physicians. Consistently with the outcome in our case, pre-operative physical therapy reported positive effects in reducing post-AAA surgery complication risks [13]. Thus, conservative treatments may need to be considered prior to surgical treatment.

Post-surgery care is important for patients with AAA and low back pain. For example, cardiovascular exercise has positive effects in reducing cardiovascular risk and suppression of AAA diameter progression [14]. Given the biomechanical impacts in various aspects, chiropractic manipulation manages complicated lumbar disc herniation even after the AAA treatment [15–17]. In addition, a combination with acupuncture therapy may provide an improved outcome [18], consistently with the present case. This case also suggests that conservative treatments for low back pain can be performed for patients with AAA without causing any complications, consistently with the lack of literature showing any adverse effect of chiropractic spinal manipulation on AAA [19]. Although it contrasts with the traditional belief that AAA is a contraindication to spinal manipulation [7, 8], the current evidence supports that conservative management is sometimes effective in managing patients with surgical advice [20].

## CONCLUSION

This case supports that low back pain in patients with AAA can be managed by conservative treatment options without any complications. More case reports of AAA patients with low back pain are warranted to assess the effectiveness and safety of manual therapy along with surgical treatment for AAA.

## Data Availability

Further data is available from the corresponding author on reasonable request.
